# Mechanisms of Resistance to Tyrosine Kinase Inhibitors in Myeloid Leukemias

**DOI:** 10.7759/cureus.86322

**Published:** 2025-06-18

**Authors:** Yazeed Alekrish, Salman Alotaibi, Zafar Iqbal, Aamer Aleem

**Affiliations:** 1 Department of Medicine, College of Medicine, King Saud University, Riyadh, SAU; 2 Department of Clinical Laboratory Sciences, College of Applied Medical Sciences, King Saud bin Abdulaziz University for Health Sciences, National Guard Health Affairs, Al Ahsa, SAU

**Keywords:** acute myeloid leukemia, chronic myeloid leukemia, mechanism, myeloid leukemias, resistance, tyrosine kinase inhibitors

## Abstract

Tyrosine kinase inhibitors (TKIs) have transformed outcomes in chronic myeloid leukemia (CML) and FLT3-mutated acute myeloid leukemia (AML), yet durable remissions are curtailed by the emergence of drug resistance. This review summarizes the principal mechanisms that underlie that resistance.

In CML, the most common mechanism is the development of point mutations in the BCR::ABL1 kinase domain (KD). Additional layers of resistance arise when imatinib, a substrate for the P-glycoprotein (P-gp) efflux pump, is shunted out of the intracellular space and when leukemic cells engage alternative signaling pathways such as the SIRT1 and JAK2-STAT5. Up-regulation of the WNT/β-catenin pathway and epigenetic changes such as HOXA4 and PDLIM4 promoter hypermethylation have likewise been linked to TKI resistance.

FLT3-mutated AML shows a parallel yet distinct pattern. One of the most common mechanisms of acquired resistance to FLT3 inhibitors is point mutations in FLT3 itself; the gatekeeper F691L, N676K and K429E substitutions cause resistance to clinically used FLT3 inhibitors. Resistance is also driven by activation of alternative signaling cascades: RAS/MAPK and IDH2-associated pathways frequently emerge and make FLT3 inhibition less effective. After initial therapy, clonal selection allows inhibitor-insensitive subclones to dominate, while bone-marrow stromal factors, high CYP3A4 activity together with FGF2/FGFR1-mediated MAPK signaling, protect blasts from FLT3 inhibitors.

It is important to study the mechanisms of resistance responsible for treatment failure to develop therapeutic strategies to overcome this resistance. This paper aims to review the important mechanisms of resistance to TKIs, both in CML and AML.

## Introduction and background

Hematological malignancies (HM) are one of the common type of cancers and include a wide variety of disorders, which are highly heterogeneous and can affect all ages. Among them, leukemias comprise a significant proportion of the HM and are further divided into myeloid and lymphoid lineages, each of which has acute and chronic subtypes. Further subdivisions are based on specific cellular types and genetic characteristics [1].

Myeloid leukemias (ML) are clonal disorders, which arise from the hematopoietic stem cells or myeloid progenitors in the bone marrow (BM). The annual incidence of chronic myeloid leukemia (CML) in the United States is around 4800 cases [2], while the incidence of acute myeloid leukemia (AML) being around 23,000 cases per year [3]. However, it is worth noting that the prevalence of these conditions is trending up over the years, particularly CML, which is likely due to improvements in treatment strategies and increased survival of these patients [2]. With the introduction of tyrosine kinase inhibitors (TKIs) to target BCR::ABL1, the overall survival (OS) of CML patients has markedly improved as demonstrated by multiple studies and real-world data [4,5]. In AML, different types of TKIs, such as FMS-like tyrosine kinase 3 (FLT3) inhibitors, have shown efficacy in FLT3-mutated AML and have improved the outcome of some types of AML, including patients with FLT3 mutations [6-8].

Rationale and objective

Although the introduction of TKIs has been a breakthrough and a major step forward in the management of ML, particularly CML, it is important to note that some patients may not respond to TKIs, or relapse and progress after initial response, as some leukemia cells could develop resistance to these drugs, rendering them ineffective. The resistance to therapy can be either primary (patient not responding to treatment from the beginning) or secondary (patient relapsing after a period of response to the therapy). Numerous studies have been carried out to investigate the mechanisms of resistance of leukemia cells to these drugs and the strategies and treatment approaches likely to be effective to prevent the development of resistance. This paper aims to review the important resistance mechanisms to TKI therapy for the management of ML.

## Review

Tyrosine kinase inhibitors

TKIs are therapeutic agents that target specific protein kinases and signaling pathways. They are used in the management of many types of cancers propagated by increased activity of protein tyrosine kinases [9]. They target and inhibit specific protein kinases, including prototypic oncogenes that play an essential role in the development of many malignancies, such as BCR::ABL1, EGFR, and VEGFR [10].

Tyrosine Kinase Inhibitor Uses in Chronic Myeloid Leukemia

TKIs targeting the BCR::ABL1 fusion protein are used in the treatment of CML and have revolutionized the management of CML. TKIs approved to treat CML include imatinib mesylate (IM), dasatinib, nilotinib, bosutinib, ponatinib, and asciminib [4]. These are oral agents and constitute the cornerstone of CML management [4,10]. Despite the excellent outcomes, some patients might not respond to some of these agents or develop secondary resistance, and may require different management strategies [11].

The introduction of IM was an important step forward in the management of CML. The IRIS study compared IM with interferon-alpha plus cytarabine in 1106 adult patients with newly diagnosed Philadelphia chromosome-positive (Ph+ve) CML in chronic phase and found that IM was significantly better in terms of cytogenetic response rates, progression-free survival, and progression to the accelerated phase or blast crisis (BC) [12]. This study led to the approval of IM for CML and the introduction of IM changed the paradigm of CML management [13].

A study conducted to estimate the trends in survival for patients with CML in Sweden analyzed the data of 2662 patients and found significant improvements in the long-term survival, particularly among younger patients [14]. This study demonstrated that improvement in survival rates was most likely attributable to advances in the management strategies for CML. The improvement in survival rates was more pronounced among younger CML patients in the earlier studies with a modest improvement in the older age groups [15]. Subsequently, multiple studies have reported that TKI therapy is also effective in older patients. One study conducted on 423 patients with Ph+ve CML treated with IM aimed to shed light on whether the previously observed poor prognosis in older CML patients was related to intrinsic worse disease biology or treatment-associated factors. This study found that older patients with CML were less likely to receive IM and that age disparities in the treatment with IM likely contributed to worse survival for many elderly CML patients [16]. Therefore, it appears that the previously observed less favorable prognosis in older CML patients treated with IM was related to treatment-associated factors rather than intrinsic disease biology [13,16].

Tyrosine Kinase Inhibitor Uses in Acute Myeloid Leukemia

A systematic review conducted to study the effectiveness and safety of TKIs in treating AML included 2656 patients and concluded that TKIs could have a promising role in the management of AML [17]. TKIs in combination with standard induction chemotherapy have led to improved complete remission rates and OS in some types of AML [18,19]. In FLT3-mutated AML patients, first-generation TKI monotherapy produced suboptimal results in the relapsed/refractory setting; however, more potent second-generation TKIs demonstrated significant activity as single agents, and some of these agents like gilteritinib have shifted the treatment options toward chemotherapy-free approach for relapsed/refractory FLT-3-mutated AML patients [20-22].

In newly diagnosed FLT3-mutated AML patients, first- and second-generation FLT3 inhibitors (such as midostaurin and quizartinib) have shown clinical efficacy [18,19]. In contrast, in relapsed/refractory AML, second-generation FLT3 inhibitors such as gilteritinib, quizartinib, and crenolanib appear to be more active [20-22]. The difference between the two lies in that first-generation FLT3 inhibitors have more off-target effects and less potency compared to second-generation FLT3 inhibitors, which were developed to have more anti-FLT3 activity and less off-target effects [20-22]. The RATIFY trial was a phase 3 clinical study to assess whether midostaurin, in addition to standard chemotherapy, could improve OS in AML patients with a FLT3 mutation. This study randomized 717 patients either to midostaurin or a placebo, in addition to standard chemotherapy, and demonstrated that OS was better in the midostaurin arm than in the placebo arm, indicating that adding midostaurin to standard chemotherapy could improve survival in AML patients with a FLT3 mutation [18]. QuANTUM-First was a randomized, double-blind, placebo-controlled, phase 3 trial that compared quizartinib plus chemotherapy in newly diagnosed patients with FLT3 internal tandem duplication (ITD)-duplication-positive AML [19]. This trial showed significantly improved survival in the quizartinib arm and led to the approval of this drug by US FDA for newly diagnosed FLT3 ITD-positive AML, increasing the treatment options for this group of patients [19].

The results of early clinical studies in newly diagnosed or relapsed/refractory FLT3-mutated AML patients using second-generation FLT3 inhibitors like gilteritinib in combination with other agents are encouraging, and if confirmed in larger studies, may further improve the outcome of this patient population [23].

Mechanisms of resistance to tyrosine kinase inhibitors in chronic myeloid leukemia

Although development of TKIs has led to a remarkable improvement in the survival rates of CML, there is still a subset of patients who fail to respond to TKI therapy or relapse after initial response [24,25]. Although, multiple mechanisms of resistance to TKI therapy have been studied, not all of these molecular mechanisms may be clinically relevant in an individual patient [26-28].

Resistance to IM and other TKIs can be divided into either primary (i.e., innate resistance) or secondary (i.e., acquired resistance); for example, patients with primary resistance do not respond to TKI therapy from the beginning, while patients with secondary resistance respond to therapy initially, then stop responding, with relapse or progression of disease [25].

BCR::ABL1 Kinase Domain Mutations

In CML, the most common mechanism of resistance to IM and other TKIs is the development of point mutations in the BCR::ABL1 kinase domain (KD) [29]. Incidence of KD mutations has been extensively studied in IM-resistant patients, and KD mutations were found to be present in varying frequency in different studies ranging from less than one-third to more than two-third of imatinib-resistant patients [30-36]. In some studies, KD mutations were more likely to be found in IM-resistant patients with clonal cytogenetic evolution compared to those without clonal evolution [32] and in patients with advanced phases of disease [33].

However, as demonstrated by various studies, several IM-resistant patients do not test positive for BCR::ABL1 KD mutations [29,32,34]. The observations that some patients with IM-resistance do not harbor KD mutations indicate that different mechanisms might be implicated in IM- and TKI-resistant CML. Patients with BCR::ABL1-positive CML with IM resistance usually respond to second- or third-generation TKIs such as dasatinib, nilotinib, or ponatinib, but this depends on the patient characteristics and the type of KD mutation present [30,31,35-37].

Overexpression of Drug Efflux Pumps

IM is a substrate for P-glycoprotein (P-gp) that acts as an efflux pump moving its substrate out of the intracellular space, reducing the effectiveness of IM [38-40]. Overexpression of drug efflux pumps could be one of the mechanisms by which leukemic cells develop resistance to IM. This was demonstrated in a study performed on resistant leukemic cells and found the levels of P-gp to be higher in imatinib-resistant cells [41]. These findings were also consistent with a case-control study that aimed to measure P-gp expression levels in lymphocytes from 59 CML patients and correlate the levels with response to IM treatment. This study found that overexpression of P-gp in lymphocytes was significantly associated with poor response to IM therapy [42]. These data suggest that overexpression of P-gp may have an important role in acquired resistance to IM in CML.

Activation of Alternative Signaling Pathways

Some signaling pathways contribute to and enhance leukemic cell resistance to TKIs such as the SIRT1 pathway. The role of SIRT1 was demonstrated in a study in CML mouse models and showed that when SIRT1 was deleted in these mice, it impaired the leukemia development, and TKIs were more effective in SIRT1-deleted mice [43].

The presence of alternative signaling pathways that contribute to the survival and proliferation of leukemic cells has been demonstrated in mouse models. Recart et al. investigated the molecular mechanisms that lead to progression of CML in mice, particularly focusing on the JAK/STAT signaling pathway [44]. This study revealed that the blast phase leukemic cells have higher activity of specific signaling pathways, namely BCR::ABL1, JAK2, and STAT5A. This suggests that all of these pathways could play a role in the progression of leukemia and could be a target for future treatment combinations. It also highlights that even if one of these pathways such as BCR::ABL1 is inhibited, leukemic cells could still survive and may proliferate through other pathways, which could be a mechanism of resistance to BCR::ABL1 TKIs [44,45].

The above study also investigated whether CML stem cells in both mouse models and in cells derived from patients depend on BCR::ABL1 kinase activity for survival and showed that CML stem cell survival was not dependent on BCR::ABL1 activity as demonstrated by the survival of these cells even when BCR::ABL1 was inhibited [44]. This highlights the need for further studies to develop treatment strategies that target the kinase-independent mechanisms of resistance to BCR::ABL1 TKIs.

Epigenetic Changes

Some of the less investigated mechanisms to BCR::ABL1 TKI resistance are epigenetic changes such as HOXA4 promoter hypermethylation, which could lead to IM resistance in CML patients. A case-control study conducted on 57 IM-resistant CML patients and 38 responders found significantly higher levels of HOXA4 hypermethylation in IM-resistant patients [46].

However, it is worth noting that hypermethylation of HOXA4 is not the only gene involved in epigenetic mechanisms of resistance to imatinib (IM). A study conducted on 120 CML patients with IM resistance or intolerance, as well as those in the accelerated and blast phases of CML, revealed a significantly higher number of methylated genes in patients resistant or intolerant to IM and in those with advanced disease phases [47]. This study also found a significant association between the methylation of the PDLIM4 gene and shortened survival, regardless of the disease stage or IM responsiveness [47].

Wingless and Int-1 Pathway Up-Regulation

The WNT (Wingless and Int-1) signaling pathway is critical for the development and homeostasis of tissues through regulation of their endogenous stem cells. Abnormal WNT signaling has been described as an important mechanism in the initiation and maintenance of many cancers, via their effect on cancer stem cells. A study investigating whether gene expression changes influence the outcomes of CML patients receiving TKI therapy performed gene expression analysis at diagnosis and after six months of treatment in 11 CML patients [48]. This study found a significant up-regulation of multiple pathways (WNT/BETA-CATENIN, JAK-STAT, and POLYCOMB), with WNT pathway being the most significant for TKI resistance, as an inverse association was observed between the degree of WNT pathway up-regulation and the event-free survival of patients [48].

Table 1 presents selected studies investigating the mechanisms of resistance to TKIs in CML, along with a summary of the types of resistance mechanisms examined.

**Table 1:** Selected studies investigating mechanisms of resistance to TKIs in CML, with a summary of the types of resistance mechanisms examined CML, chronic myeloid leukemia; TKIs, tyrosine kinase inhibitors; KD, kinase domain; P-gp, P-glycoprotein; WNT, Wingless and Int-1

Reference	Study design	Sample size	TKIs involved	Results and conclusions
Muller et al. [30]	Clinical trial	805 CML patients	Imatinib	63 different BCR::ABL1 mutations detected in 48% of the imatinib-resistant or intolerant cohort
Huges et al. [31]	Clinical trial	281 CML patients	Imatinib	BCR::ABL1 mutations found in 55% of the imatinib-resistant patients
Marcé et al. [32]	Cohort	45 CML patients	Nilotinib, dasatinib	ABL mutations detected in 14 (31%) of imatinib-resistant patients. Mutations more commonly found in IM-resistant patients with clonal cytogenetic evolution
Erbilgin et al. [34]	Cohort	17 CML patients	Imatinib, nilotinib, dasatinib	BCR::ABL1 KD mutations detected in 59% of the TKI-resistant cohort
Tadesse et al. [35]	Cross-sectional	31 CML patients	Imatinib	Mutations detected in imatinib-resistant patients: non-P-loop mutations 36%, P-loop mutations 13%, alternatively spliced BCR-ABL variants 23%
Widmer et al. [38]	Experimental	MDR1^+^ and MDR1^-^ LLC-PK1 cells (transfected porcine kidney epithelial cells)	Imatinib	Imatinib efflux from cancer cells by the drug transporter Pgp among the mechanisms of imatinib resistance
Dai et al. [40]	Experimental	Mouse model	Imatinib	STI-571 (imatinib) is a substrate for P-gp. The inhibition of P-gp affects the transport of STI-571
Peng et al. [41]	Experimental	Imatinib-resistant human CML cell line (K562-imatinib)	Imatinib	Overexpression of P-gp may play a key role in acquired resistance to imatinib as shown in CML K562 imatinib cells
Ammar et al. [42]	Retrospective case‐control study	59 CML patients	Imatinib	Overexpression of P‐gp in lymphocytes significantly correlated with the failed molecular response to imatinib in CML patients
Abraham et al. [43]	Experimental	Mouse model	Nilotinib	SIRT1 promotes leukemic hematopoiesis and mediates TKI resistance
Recart et al. [44]	Experimental	Mouse model	Dasatinib	BCR::ABL1, JAK2, and STAT5A are all involved in progression of CML. Even if one pathway is suppressed, leukemic cells can still proliferate through other signaling pathways
Elias et al. [46]	Experimental	95 CML patients	Imatinib	Promoter hypermethylation of HOXA4 gene could be an epigenetic mechanism mediating imatinib resistance in CML
Jelinek et al. [47]	Experimental	120 CML patients	Imatinib	Aberrant DNA methylation is associated with resistance to imatinib
Grassi et al. [48]	Cohort	11 CML patients	Imatinib, nilotinib, dasatinib	WNT pathway mostly responsible for the TKIs resistance

Mechanisms of resistance to tyrosine kinase inhibitors in acute myeloid leukemia

The use of TKIs in AML depends on the subtype of AML and the type of mutation(s) present in each individual patient. The most commonly used TKIs in AML are FLT3 inhibitors such as midostaurin and gilteritinib, targeting FLT3 mutations. However, the development of resistance remains a challenge, as the response duration is relatively short when used as monotherapy in the relapsed/refractory setting [17,49,50]. This section will focus on mechanisms of resistance to FLT3 inhibitors in AML.

Point Mutations in Target Kinases

One of the most common mechanisms of acquired resistance to FLT3 inhibitors in AML is point mutations in the FLT3 gene. These mutations change the FLT3 protein structure in a way that makes FLT3 inhibitors unable to inhibit it, making them less effective or ineffective. FLT3 tyrosine KD mutations are detected in 7-11% of patients with AML at the time of diagnosis and can be associated with primary resistance to FLT3 inhibitors [51,52]. However, these mutations are more commonly acquired and cause secondary resistance [50,53-58].

Multiple acquired mutations conferring resistance to FLT3 inhibitors have been reported. These include the gatekeeper F691L mutation, which causes resistance to all clinically used FLT3 inhibitors [50,55]; the FLT3 N676K mutation, which confers resistance to midostaurin [57]; and the K429E mutation, which imparts resistance to crenolanib [58].

Activation of Alternative Signaling Pathways

Activation of alternative signaling pathways can make FLT3 inhibitors less effective and some of these pathways constitute important mechanisms of resistance to FLT3 inhibitors in AML. NRAS and KRAS are part of the RAS family of oncogenes, which play an important role in signaling pathways that contribute to the control of cell growth and survival. Mutations in this gene can cause abnormal activation of this pathway, leading to uncontrolled cellular proliferation that could cause AML cells to develop resistance to crenolanib, a FLT3 inhibitor [58]. Additionally, the IDH2 (isocitrate dehydrogenase 2) mutations could alter the cellular metabolism and epigenetic regulation mechanisms, which could lead to the development of an alternative oncogenic pathway that promotes the survival of cancer cells independent of FLT3 pathway, making these cells resistant to FLT3 inhibitors [59].

One study used next-generation sequencing on pre-treatment and relapsed BM samples of FLT3-mutated AML patients treated with FLT3 inhibitors to investigate the mechanisms of resistance. This study showed that RAS/MAPK signaling pathway and FLT-D835 mutations emerged following FLT3 inhibitors, and patients with emergent mutations at relapse had significantly worse OS [60]. Another study that employed whole exome sequencing (WES) on AML patient samples before and after crenolanib treatment to investigate resistance mechanisms found that this FLT3 inhibitor induced mutations in NRAS and IDH2 genes, which activate alternative signaling pathways [58].

Schmalbrock et al. analyzed peripheral blood and BM samples with WES from 54 patients to study the underlying resistance mechanisms in FLT3-ITD mutated AML. They found that around 46% of patients treated with midostaurin became FLT3-ITD negative but developed mutations in signaling pathways, providing a mechanism for resistance. In 32% of patients, no FLT3-ITD mutational change was observed, suggesting that resistance mechanisms may bypass FLT3 inhibition or that midostaurin drug levels were inadequate [54].

Another study involving 41 AML patients treated with gilteritinib found that 36% developed treatment-emergent RAS/MAPK pathway mutations, predominantly involving NRAS. These findings highlight that RAS/MAPK pathway activation can mediate resistance to gilteritinib [61].

Clonal Selection

After initial treatment with chemotherapy and a FLT3 inhibitor, FLT3-mutated clones sensitive to FLT3 inhibitors are eliminated, while those with wild-type FLT3 may survive. Reduced anti-leukemic effect has been shown in both in vivo and in vitro studies in AML cells that express both mutated and wild-type FLT3 [62,63].

In addition, leukemic subclones that survive initial treatment may re-emerge in greater numbers at relapse compared to the time of initial diagnosis, and clonal selection of more primitive leukemic clones may render them resistant to further therapy [64]. It is important to emphasize that FLT3 status should be reassessed at relapse as it may change from the initial diagnosis, which may make FLT3 inhibitors less effective.

Bone Marrow Stromal Factors

FLT3-ITD-positive blasts in the peripheral blood are rather easily eradicated by FLT3 inhibitors, but BM blasts are more difficult to eradicate and may survive FLT3 treatment [65]. Earlier FLT3 inhibitors, such as lestauritinib, had poor BM penetration and did not eliminate BM FLT3-mutated blasts [66,67].

BM stromal cells have a high concentration of CYP3A4, providing protection to FLT3-ITD-positive leukemia cells [68]. Some FLT3 inhibitors are metabolized by CYP3A4 enzymes, and the therapeutic activity of gilteritinib and sorafenib has been reported to be affected by BM stromal cell-mediated CYP3A4 metabolism [69].

Furthermore, the BM stroma seems to protect blasts from FLT3 inhibitors through extracellular receptor kinase-mediated signaling. FGF2 is a growth factor that activates fibroblast growth factor receptor 1 (FGFR1), expressed on AML blasts, to promote downstream MAPK signaling independent of FLT3 [65], and has been implicated in resistance to quizartinib [70].

Table 2 highlights the mechanisms of resistance to TKIs in AML and a summary of resistance mechanism(s) involved.

**Table 2:** Mechanisms of resistance to FLT3 inhibitors in AML and summary of involved resistance mechanisms AML, acute myeloid leukemia; TKIs, tyrosine kinase inhibitors

Authors	Study design	Sample	TKI(s)	Results
Williams et al. [49]	Experimental	Mouse model	Lestaurtinib, sorafenib, midostaurin, sunitinib	F621L, A627P, F691L, and Y842C mutations in FLT3/ITD confer varying levels of resistance to FLT3 inhibitors
Schmalbrock et al. [54]	Clinical trial	54 AML patients	Midostaurin	FLT3-ITD persistence can be caused by selection of resistant FLT3 clones, mechanisms bypassing FLT3 inhibition or insufficient drug activity
Sharzehi et al. [55]	Experimental	Cell cultures	Gilteritinib	FLT3 F691L gatekeeper mutation promotes clinical resistance to gilteritinib + venetoclax in AML
Smith et al. [56]	Experimental	Single cell analysis from AML patients	Quizartinib	FLT3-ITD^+^ cells without FLT3 D835 mutations are functionally resistant to quizartinib
Heidel et al. [57]	Experimental	6 AML patients	PKC412 (midostaurin)	FLT3-ITD-N676K mutation confers an intermediate level of resistance to PKC412 in vitro
Zhang et al. [58]	Experimental	Whole exome sequencing of AML patients	Crenolanib	Subclonal RAS mutations and concomitant TET2 mutations contribute to crenolanib resistance
Alotaibi et al. [60]	Experimental	106 AML patients with primary TKI resistance, 67 AML patients with secondary resistance	Midostaurin, sorafenib, gilteritinib, quizartinib, crenolanib	Higher frequencies of pretreatment, cohort-level variant allelic frequencies of RAS mutations were likely associated with primary resistance in patients treated with type I FLT3 inhibitors. RAS/MAPK and FLT3-D835 mutations most commonly emerged at relapse following both type I and type II FLT3 inhibitor-based therapies.
McMahon et al. [61]	Cohort	41 AML patients	Gilteritinib	RAS/MAPK pathway activation mediates resistance to gilteritinib

Table 3 gives the names of the mechanisms and pathways involved in TKI resistance in CML and AML. 

**Table 3:** Main pathways and mechanisms of TKI resistance involved in CML and AML AML, acute myeloid leukemia; CML, chronic myeloid leukemia; TKIs, tyrosine kinase inhibitors; WNT, Wingless and Int-1

	Mechanisms of resistance to TKIs in CML
1	BCR::ABL1 kinase domain mutations
2	Overexpression of drug efflux pumps
3	Activation of alternative signaling pathways
4	Epigenetic changes
5	WNT pathway up-regulation
	Mechanisms of resistance to FLT3 inhibitors in AML
1	Point mutations in target kinases
2	Activation of alternative signaling pathways
3	Clonal selection of wild type or resistant FLT3 clones
4	Bone marrow stromal factors contributing to poor FLT3 inhibitor penetration or increased clearance

Clinical implications of resistance to tyrosine kinase inhibitors and strategies to overcome resistance

Even though the development of TKIs have been a tremendous step forward in the management of several malignancies, many patients still develop resistance to these drugs. Multiple strategies have been tried to overcome this issue, including combining TKIs with other targeted therapies, such as PI3K/AKT/mTOR pathway inhibitors in CML [71]. A study conducted to compare the response of BC CML leukemic stem cells (LSC) to JAK2 inhibitors, alone and in combination with a BCR::ABL1 inhibitor in reducing the leukemic burden and impairing the self-renewal capability of BC LSC, found that combination of a JAK2 inhibitor (SAR302503) and a BCR::ABL1 inhibitor (dasatinib) was significantly more effective in impairing the ability of LSC to renew themselves and reducing the number of LSC, indicating this strategy may be important in preventing the leukemia from developing resistance and relapsing [45].

CML patients who develop resistance to multiple TKIs need alternative therapies. Omacetaxine mepesuccinate (previously known as homoharringtonine) is a natural alkaloid with anticancer activity partly through inhibition of protein synthesis and induction of apoptosis [72-75]. Although, less commonly used, it has significant activity in CML, particularly against T315I mutation. Ponatinib is useful after resistance to second-generation TKIs and is active against T315I mutation but its cardiovascular (CV) toxicity makes it a less favorable option, particularly for patients with existing CV disease or at risk of developing CV morbidity [76]. With the recent approval of asciminib for CML patients resistant to two or more TKIs or patients with T315I mutation, the treatment options for CML patients with resistance have increased [77]. For patients who develop resistance to multiple TKIs, allogenic hematopoietic stem cell transplantation (AHSCT) remains an important strategy and potential curative option in eligible patients, particularly those with advanced phases of CML [78,79]. The decisions for the next line of therapy should be based on specific considerations in each individual patient such as age, comorbidities, and presence of a particular KD mutation [78,79].

Currently, three FLT3 inhibitors are approved for the treatment of AML: midostaurin and quizartinib, each in combination with chemotherapy for newly diagnosed FLT3-mutated AML, and gilteritinib as a single agent for relapsed or refractory FLT3-mutated AML [18,19,22]. Patients who develop resistance to these agents have limited options [80], and apart from the experimental therapies, AHSCT may be able to offer leukemia control but available only for a limited number of eligible patients.

Potential future strategies to overcome FLT3 inhibitor resistance in AML include the development of novel small-molecule FLT3 inhibitors, combination therapies involving FLT3 inhibitors, and the design of multi-target inhibitors [81-84]. Understanding the FLT3 receptor sites most susceptible to resistance-conferring mutations has guided the development of new inhibitors capable of overcoming these resistances [85-88], which may further improve patient outcomes.

## Conclusions

Although this review is not exhaustive, it highlights the key mechanisms of resistance to TKIs in CML and AML in a simplified manner, providing a broad overview of the topic. Resistance mechanisms to TKIs in HMs are diverse, with point mutations in target kinases being the most common. Understanding these resistance pathways is crucial for identifying therapeutic targets and developing more effective treatment strategies to improve patient outcomes. Future research should focus on exploring combination therapies to overcome resistance, as well as investigating additional mechanisms that may serve as novel therapeutic targets.
